# FDMNet: A Multi-Task Network for Joint Detection and Segmentation of Three Fish Diseases

**DOI:** 10.3390/jimaging11090305

**Published:** 2025-09-06

**Authors:** Zhuofu Liu, Zigan Yan, Gaohan Li

**Affiliations:** The Higher Educational Key Laboratory for Measuring and Control Technology and Instrumentations of Heilongjiang Province, Harbin University of Science and Technology, Harbin 150080, China

**Keywords:** fish disease detection, lesion segmentation, multi-task network

## Abstract

Fish diseases are one of the primary causes of economic losses in aquaculture. Existing deep learning models have progressed in fish disease detection and lesion segmentation. However, many models still have limitations, such as detecting only a single type of fish disease or completing only a single task within fish disease detection. To address these limitations, we propose FDMNet, a multi-task learning network. Built upon the YOLOv8 framework, the network incorporates a semantic segmentation branch with a multi-scale perception mechanism. FDMNet performs detection and segmentation simultaneously. The detection and segmentation branches use the C2DF dynamic feature fusion module to address information loss during local feature fusion across scales. Additionally, we use uncertainty-based loss weighting together with PCGrad to mitigate conflicting gradients between tasks, improving the stability and overall performance of FDMNet. On a self-built image dataset containing three common fish diseases, FDMNet achieved 97.0% mAP50 for the detection task and 85.7% mIoU for the segmentation task. Relative to the multi-task YOLO-FD baseline, FDMNet’s detection mAP50 improved by 2.5% and its segmentation mIoU by 5.4%. On the dataset constructed in this study, FDMNet achieved competitive accuracy in both detection and segmentation. These results suggest potential practical utility.

## 1. Introduction

Aquaculture, a globally important food production sector, contributes to food security and promotes sustainable economic development [1,2,3]. However, as stocking density increases, fish diseases—such as bacterial hemorrhagic septicemia, saprolegniasis, and fish lice—occur more frequently and remain a significant constraint to profitability [4,5,6]. Outbreaks spread rapidly, causing large-scale mortality and secondary environmental pollution [7,8,9]. Therefore, early detection and precise lesion segmentation are critical for intelligent aquaculture systems [10].

Traditional diagnosis relies on manual sampling and visual inspection, which is subjective, time-consuming, and prone to missing the optimal treatment window. With advances in computer vision, deep learning provides alternatives to manual inspection for object detection and image segmentation in fish-health monitoring. However, many existing systems treat detection or segmentation in isolation or target only a single fish disease.

To address these gaps, we propose FDMNet, a multi-task framework built on YOLOv8. It adds a semantic segmentation branch and enables simultaneous detection and segmentation tasks. The segmentation branch employs multi-scale feature fusion to capture lesion boundaries and fine details better. FDMNet uses C2DF modules that combine channel and spatial attention to enhance segmentation of blurred boundaries and small lesions. We adopt uncertainty-based loss weighting with PCGrad for joint training to mitigate gradient conflicts between tasks and improve training stability.

The main contributions of this work are as follows:

1. We present FDMNet, a lightweight multi-task model that integrates a YOLOv8 detection head and a semantic segmentation branch to simultaneously detect and segment three fish diseases (bacterial hemorrhagic septicemia, saprolegniasis, and fish lice).
2. We adapt the C2DF module by integrating dynamic feature fusion into C2f blocks. C2DF replaces C2f modules in the neck and the segmentation branch, improving the local-detail representation and boundary modelling.
3. The study adopts a multi-task optimisation strategy that combines uncertainty-based loss weighting with PCGrad to improve training stability and reduce inter-task gradient interference.
4. This study developed a multi-disease fish image dataset and evaluated all modules used in the dataset. We compared FDMNet with Faster R-CNN, YOLOv8n, YOLOv11n, RT-DETR, YOLO-FD, and Mask R-CNN for the detection task. We compared FDMNet with U-Net, DeepLabv3-ResNet50, DeepLabv3+-ResNet50, YOLO-FD and Mask R-CNN for the segmentation task. On our dataset, FDMNet achieved competitive performance on both tasks, aligning with the needs of image-based fish disease diagnosis.

This paper is organised as follows. Section 2 reviews related work on fish disease detection and lesion segmentation. Section 3 introduces our self-constructed dataset, then describes the overall structure of FDMNet and introduces the C2DF block and the multi-scale semantic segmentation branch. It also describes a training strategy that combines uncertainty weighting with PCGrad. Section 4 details the training configuration and specifies the evaluation metrics. It then reports the experimental results. These include detection comparisons with Faster R-CNN, YOLOv8n, YOLOv11n, RT-DETR, YOLO-FD, and Mask R-CNN, segmentation comparisons with U-Net, DeepLabv3, DeepLabv3+, YOLO-FD, and Mask R-CNN, ablation studies, and Grad-CAM visualisation. Section 5 discusses the main findings, notes model and data limitations, and outlines future work. Section 6 presents the conclusions.

## 2. Related Work

Research on fish disease image analysis has progressed from classical machine-learning pipelines to deep learning approaches. Classical machine learning pipelines combined pre-processing, feature extraction, and a classifier [11]. Early methods relied on handcrafted features with simple classifiers, which improved efficiency over manual inspection. Ahmed et al. reported an SVM-based approach using image enhancement, k-means clustering, and texture features, achieving 94.1% accuracy on a salmon-disease image dataset with augmentation [12]. Waleed et al. investigated RGB, YCbCr, and XYZ colour spaces with Gaussian modelling to segment infected regions [13]. However, the reliance on manual feature design made adaptation and maintenance increasingly demanding as datasets and task complexity grew. With the advent of convolutional neural networks, two main lines have emerged: detection models that localise diseased fish and segmentation models that delineate lesions.

Building on this shift, advances in CNN-based methods have further accelerated fish disease image analysis [14]. In detection, Yu et al. proposed a YOLOv4-MobileNetV3 variant for identifying four deep-sea fish diseases and reported low latency on their dataset [15]. Hamzaoui et al. introduced FishDETECT, which reported high precision in low-light conditions using transfer learning and tailored optimisation [16]. Zhou et al. designed a YOLOv7-based feature-enhancement module and reported accuracy and speed improvements for static underwater imagery [17]. Li et al. presented DDEYOLOv9 with the DRNELAN4 module and a dynamic-convolution head, improving abnormal behaviour detection in their setting [18]. Cai et al. proposed NAM-YOLOv7, which combines Auto-MSRCR enhancement, NAM attention in ELAN, and MPDIoU loss [19]. They reported 97.3% accuracy, 93.8% recall, and approximately 0.18 s per image, outperforming several YOLO-series baselines for SVC symptom localisation.

In segmentation, Zhang et al. proposed an iterative instance segmentation mechanism combining YOLOv5 and YOLOv8, which reported strong performance on the DeepFish dataset [20]. Long et al. enhanced YOLOv5s with Coordinate Attention and CBAM, plus positive-sample matching and EMA, and reported 95.88% mAP and improvements over YOLOv3, YOLOv4, YOLOv5s, YOLOv5m and SSD on their dataset [21]. Ben Tamou, Benzinou, and Nasreddine explored ResNeXt-101 transfer learning with targeted augmentation and a hierarchical family-to-species classifier, reporting 99.86% accuracy on FRGT and 81.53% on LifeClef-2015 [22]. Li et al. improved DeepLabv3+ with adaptive-threshold pre-processing and reported 92.6% mIoU on Fish4Knowledge [23]. Kong et al. proposed AASNet with Linear Correlation Attention and Dynamic Adaptive Focal Loss, reporting an mAP of 47.4 on USIS and 28.9 ms inference time [24]. Akram et al. proposed an autonomous net-pen defect detector using a multi-scale semantic-segmentation topology that fuses attention across decomposition levels and reported gains of 6.58%, 3.69%, 6.44%, and 4.78% mAP on LABUST, KU, NDv1, and NDv2, respectively [25]. Paul et al. introduced FishSegSSL, a semi-supervised fisheye image segmentation method with pseudo-label filtering and dynamic thresholding, reporting a 10.49% improvement over supervised baselines [26]. To address both tasks simultaneously, Li et al. proposed YOLO-FD, integrating a segmentation branch into a YOLO detector for fish disease analysis [27].

Although these studies have advanced aquaculture image analysis, most remain limited to a single task. The multi-task model YOLO-FD detects only a single disease and cannot accommodate additional categories. Moreover, its segmentation performance can be further improved. These limitations motivate the development of a unified multi-task framework that couples detection with pixel-level lesion segmentation, enabling coverage of more fish disease categories and improving both detection and segmentation performance.

## 3. Materials and Methods

### 3.1. Dataset Acquisition

**Figure 1:**
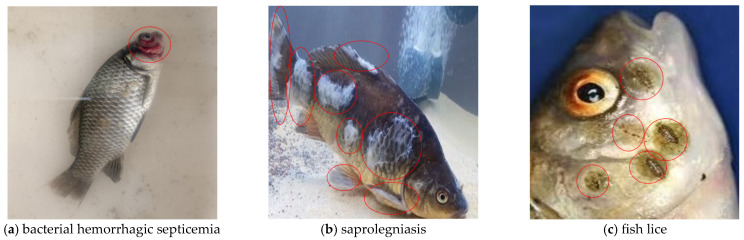
Lesion characteristics of bacterial hemorrhagic septicemia, saprolegniasis, and fish lice.

The image dataset used in this study was obtained from laboratory-reared fish and field aquaculture environments, covering three common freshwater fish diseases: bacterial hemorrhagic septicemia, saprolegniasis, and fish lice. As shown in Figure 1, bacterial hemorrhagic septicemia typically presents with congestion or haemorrhaging in areas such as the mouth, gills, jaws, eyes, and fins. Saprolegniasis is characterised by white, cotton-like mycelial growths on the body surface. Fish lice are ectoparasites that attach to the fish’s skin, exhibiting a semi-transparent or light brown appearance, with some species presenting as black spots or striped patterns.

This study collected images of healthy and diseased fish above and below the water surface using a smartphone with high-resolution imaging capabilities. Data augmentation techniques were applied to expand the dataset, followed by rigorous screening and cleaning to retain only high-quality images for experimentation to enhance the model’s generalisation ability. The final dataset comprised 2148 images, including 528 images of bacterial hemorrhagic septicemia, 512 saprolegniasis, 508 fish lice, and 600 healthy fish. We split the dataset into training, validation, and test sets in a 7:2:1 ratio. For detection, bounding boxes were annotated with LabelImg. For segmentation, masks were annotated with LabelMe. We applied the same label taxonomy in both tasks: bacterial hemorrhagic septicemia as “red,” saprolegniasis as “sap,” and fish lice as “lice.” Table 1 displays the image counts by class and data split (train/validation/test) for bacterial hemorrhagic septicemia, saprolegniasis, and fish lice. It also lists the overall totals of detection bounding boxes and segmentation masks.

### 3.2. Overall Structure of FDMNet

**Figure 2:**
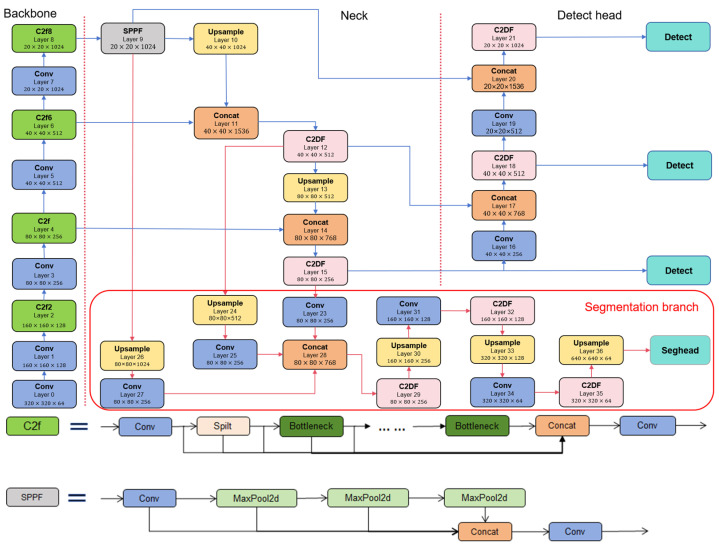
Overview of FDMNet. Each module is labelled by its layer number. Tensor dimensions are annotated beneath modules as height (H) × width (W) × channels (C). Insets show the internal structures of the C2f block and the SPPF module.

We adopt YOLOv8n as the base model because of its established architecture, modular design, and mature deployment ecosystem. However, standard YOLOv8 follows a single-task paradigm. We therefore extend YOLOv8n with a semantic-segmentation branch and a multi-task training scheme, forming FDMNet, which jointly performs disease detection and lesion segmentation for three fish diseases. As shown in Figure 2, the entire network structure of FDMNet consists of four main components: a backbone, a neck, a detection head, and a segmentation branch. The backbone extracts multi-scale feature representations from input images. In contrast, the neck uses a feature pyramid to enhance and fuse cross-scale features. The detection head localises and classifies fish disease targets. The segmentation branch uses multi-scale feature fusion to improve granularity and delineate lesion regions at the pixel level. In this architecture, the backbone and neck jointly function as shared encoders, whereas the detection head and segmentation branch serve as task-specific decoders. Object detection operates at the image level to identify diseased fish, whereas semantic segmentation functions at the pixel level to precisely outline lesion areas. By unifying these complementary tasks within a multi-task learning framework, FDMNet enables more accurate and comprehensive diagnosis of fish diseases.

#### 3.2.1. C2DF Module

The three fish diseases—bacterial hemorrhagic septicemia, saprolegniasis, and fish lice—often show small lesion areas, indistinct boundaries, and substantial inter-class variability. These characteristics challenge both detection and segmentation. During multi-scale feature fusion, the original YOLOv8 may attenuate fine local cues, particularly near subtle boundaries and complex textures. This attenuation produces imprecise localisation and blurred masks. To address these issues, we integrate the Dynamic Feature Fusion (DFF) mechanism from MSCB-Unet [28] into the YOLOv8 C2f block, yielding a modified unit termed C2DF. As shown in Figure 2, from layer 12 onward throughout the neck, detection head, and segmentation branch, C2f is replaced by C2DF. This fusion method enables the model to dynamically enhance local details and effectively integrate global semantics while preserving the lightweight main structure, improving detection accuracy and segmentation performance.

**Figure 3:**
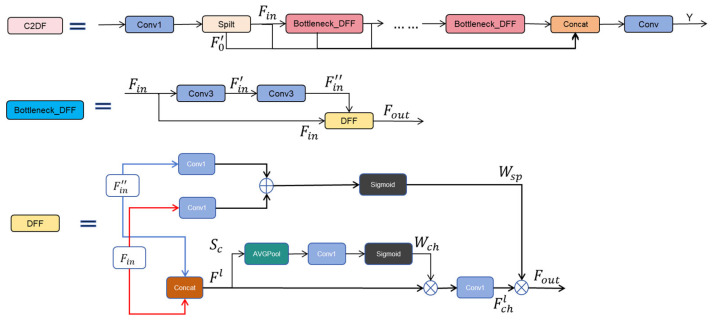
C2DF module and its internal network structure. Inset diagrams detail the internal structures of C2DF, Bottleneck_DFF and DFF.

As shown in Figure 3, the proposed C2DF module comprises the following components: input channel expansion convolution, Split operation, bottleneck_DFF modules, a Concat fusion layer, and a final output convolution.

We denote the input feature $X_{cf}\left[b,m,i,j\right]\in R^{B\times C_{in}\times H\times W}$, where $b\in \left\{1,2,\ldots \ldots ,B\right\}$ indexes the batch, $m\in \left\{1,2,\ldots \ldots ,C_{in}\right\}$ indexes the input channels, and $i\in \left\{1,2,\ldots \ldots ,H\right\},\;\;j\in \left\{1,2,\ldots \ldots ,W\right\}$ index the spatial height and width. Here, *B* is the batch size, $C_{in}$ is the number of input channels, and *H* and *W* are the spatial dimensions of the input feature map. The model outputs a feature tensor $Y_{cf}\in R^{B\times C_{out}\times H\times W}$, where $C_{out}$ represents the number of output channels, and $C_{in}=C_{out}$.

As shown in Equation (1), the input feature $X_{cf}\left[b,m,i,j\right]$ is first processed by a 1×1 convolutional layer (Conv1), which performs a pointwise channel expansion. This operation yields an intermediate feature map $\;F_{0}\left[b,k_{1},i,j\right]\in R^{B\times 2C^{\prime }\times H\times W}$, where $C^{\prime }=C_{out}\cdot e,0<e<1$, and e denotes the channel compression ratio. Here, $C^{\prime }$ is the reduced number of channels after compression. Let $W_{c1}\left[k_{1},m\right]$ denote the weight matrix of Conv1, where $k_{1}\in \left[1,2,\ldots \ldots 2C^{\prime }\right]$ indexes the output channels. The element $X_{cf}\left[b,m,i,j\right]$ is the value at sample *b*, channel *m*, and spatial position (*i*, *j*).

(1) $$F_{0}\left[b,k_{1},i,j\right]=\mathrm{Conv}1\left(X_{cf}\left[b,m,i,j\right]\right)=\overset{C_{in}}{\underset{m=1}{\sum }}W_{c1}\left[k_{1},m\right]X_{cf}\left[b,m,i,j\right]\;$$

The feature map $F_{0}\left[b,k_{1},i,j\right]$ is evenly split along the channel dimension using a Split operation, producing two sub-feature maps. One part is used as the initial residual feature, denoted as $F_{0}^{\prime }\left[b,k_{2},i,j\right]\in R^{B\times C^{\prime }\times H\times W}$. The other part denoted as $F_{in}\left[b,k_{2},i,j\right]\in R^{B\times C^{\prime }\times H\times W}$, serves as the input to the subsequent Bottleneck_DFF module.

The Bottleneck_DFF module performs deep feature extraction and adaptive fusion. It applies two sequential 3 × 3 convolutional layers (Conv3), followed by the Dynamic Feature Fusion (DFF) operation. The input $F_{in}\left[b,k_{2},i,j\right]$ is first passed through a Conv3 layer to produce $F_{in}^{\prime }\in R^{B\times C^{\prime }\times H\times W}$, which is then processed by a second Conv3 layer to yield $F_{in}^{\prime \prime }\left[b,k_{2},i,j\right]\in R^{B\times C^{\prime }\times H\times W}$. Both $F_{in}\left[b,k_{2},i,j\right]$ and $F_{in}^{\prime \prime }\left[b,k_{2},i,j\right]$ are subsequently fed into the DFF module for feature fusion, as shown in Equation (2). We denote the output channel index of each convolution operation as $k_{2}\in \left\{1,2,\ldots \ldots ,\;C^{\prime }\right\}$:

$$F_{in}^{\prime }\left[b,k_{2},i,j\right]=\mathrm{Conv}3\left(F_{in}\left[b,k_{2},i,j\right]\right)\;\;$$

(2a) $$F_{in}^{\prime }\left[b,k_{2},i,j\right]=\overset{C^{\prime }}{\underset{m=1}{\sum }}\overset{1}{\underset{u=-1}{\sum }}\overset{1}{\underset{v=-1}{\sum }}W_{c2}\left[k_{2},m,u+1,v+1\right]F_{in}\left[b,m,i+u,j+v\right]\;$$

$$F_{in}^{\prime \prime }\left[b,k_{2},i,j\right]=\mathrm{Conv}3\left(F_{in}^{\prime }\left[b,k_{2},i,j\right]\right)\;\;$$

(2b) $$F_{in}^{\prime \prime }\left[b,k_{2},i,j\right]=\overset{C^{\prime }}{\underset{m=1}{\sum }}\overset{1}{\underset{u=-1}{\sum }}\overset{1}{\underset{v=-1}{\sum }}W_{c3}\left[k_{2},m,u+1,v+1\right]F_{in}^{\prime }\left[b,m,i+u,j+v\right]\;$$

Here, $W_{c2}\left[k_{2},m,u+1,v+1\right]$ and $W_{c3}\left[k_{2},m,u+1,v+1\right]$ denote the learnable weights of the first and second 3 × 3 convolutional layers in the Bottleneck_DFF module. Where $u,v\in \left[-1,0,1\right]$ represent the relative spatial offsets of the kernel along the height and width dimensions.

The DFF module integrates both channel attention and spatial attention mechanisms, enabling dynamic recalibration of feature importance based on image content. This attention increases sensitivity to small lesion regions that are difficult to detect. Compared with static fusion, DFF offers greater adaptability and selectivity and reduces performance drops when appearance varies across disease types. In our experiments, it yields higher detection accuracy and sharper segmentation boundaries.

Equation (3) shows the processing flow of the channel attention within the DFF. Specifically, we concatenate the input features $F_{in}\left[b,k_{2},i,j\right]$ and $F_{in}^{\prime \prime }\left[b,k_{2},i,j\right]$ via the Concat to produce the fused featured map $F^{l}\in R^{B\times 2C^{\prime }\times H\times W}$. The model passes this feature map through a global average pooling layer (AVGPool), a Conv1, and a Sigmoid to generate the channel attention weights $W_{ch}$.

(3) $$F^{l}\left[b,k_{2},i,j\right]=\mathrm{Concat}\left(F_{in}^{\prime \prime }\left[b,k_{2},i,j\right],\;F_{in}\left[b,k_{2},i,j\right]\right)$$

As shown in Equation (4), the fused feature map $F^{l}\left[b,k_{2},i,j\right]$ is passed through an AVGPool to obtain the channel descriptor $S_{c}\left[b,k\right]\in R^{B\times 2C^{\prime }}$, which captures the mean activation over all spatial positions (*i*, *j*) for channel $k_{2}$.

(4) $$S_{c}\left[b,k_{2}\right]=\frac{1}{HW}\overset{H}{\underset{i=1}{\sum }}\overset{W}{\underset{j=1}{\sum }}F^{l}\left[b,k_{2},i,j\right]$$

Subsequently, the descriptor $S_{c}\left[b,k_{2}\right]$ is passed through a Conv1 with a weight matrix $W_{4}\left[k_{2},m\right]$, followed by a Sigmoid activation function to generate the final channel attention weights $W_{ch}\left[b,k_{2}\right]$. $W_{4}\left[k_{2},m\right]\in R^{C^{\prime }\times 2C^{\prime }}$ denotes the learnable kernel of the 1 × 1 convolution that maps the concatenated channel descriptor to the desired attention dimension.

$$W_{ch}\left[b,k_{2}\right]=\mathrm{Sigmoid}\left(\mathrm{Conv}1\left(S_{c}\left[b,k_{2}\right]\right)\right)=\mathrm{Sigmoid}\left(\overset{2c^{\prime }}{\underset{m=1}{\sum }}W_{4}\left[k_{2},m\right]S_{c}\left[b,m\right]\right)$$

(5) $$W_{ch}\left[b,k_{2}\right]=\frac{1}{1+\exp\left(-\sum_{m=1}^{2C^{\prime }}W_{4}\left[k_{2},m\right]S_{c}\left[b,m\right]\right)}$$

Applying the channel attention weights $W_{ch}\left[b,k_{2}\right]$ to the fused feature map $F^{l}\left[b,k_{2},i,j\right]\;$ performs a channel-wise weighting. Next, a Conv1 with a weight matrix $W_{5}\left[k_{2},m\right]\in R^{C^{\prime }\times 2C^{\prime }}$ generates the channel attention-enhanced output $F_{ch}^{l}\left[b,k_{2},i,j\right]$. The weight matrix $W_{5}\left[k_{2},m\right]$ projects the weighted features into the intermediate space. Here, ⊙ denotes element-wise multiplication. As defined in Equation (6) is

(6) $$F_{ch}^{l}\left[b,k_{2},i,j\right]=\mathrm{Conv}1\left(W_{ch}⊙F^{l}\right)=\overset{2c^{\prime }}{\underset{m=1}{\sum }}W_{5}\left[k_{2},m\right]W_{ch}\left[b,m\right]F^{l}\left[b,m,i,j\right]\;$$

We formulate the spatial attention mechanism in the DFF module as shown in Equation (7)—the Conv1 layers, with learnable weight matrices $W_{6}\left[k_{2},m\right]$ and $W_{7}\left[k_{2},m\right]$, individually process both $F_{in}\left[b,k_{2},i,j\right]$ and $F_{in}^{\prime \prime }\left[b,k_{2},i,j\right]$. The resulting feature maps are summed element-wise and passed through a Sigmoid activation function to generate the spatial attention map $W_{sp}\left[b,0,i,j\right]$. Here, $W_{6}\left[k_{2},m\right]\in R^{1\times C^{\prime }}$ and $W_{7}\left[k_{2},m\right]\in R^{1\times C^{\prime }}$ control the contribution of each spatial channel in the respective features to the final spatial weighting:

$$W_{sp}\left[b,0,i,j\right]=\;\mathrm{Sigmoid}(\mathrm{Conv}1(F_{in}\left[b,k_{2},i,j\right])+\mathrm{Conv}1\left(F_{in}^{\prime \prime }\left[b,k_{2},i,j\right]\right))\;$$

$$W_{sp}\left[b,0,i,j\right]=\mathrm{Sigmoid}\left(\overset{c^{\prime }}{\underset{m=1}{\sum }}W_{6}\left[k_{2},m\right]F_{in}\left[b,m,i,j\right]+\overset{c^{\prime }}{\underset{m=1}{\sum }}W_{7}\left[k_{2},m\right]F_{in}^{\prime \prime }\left[b,m,i,j\right]\right)$$

(7) $$W_{sp}\left[b,0,i,j\right]=\frac{1}{1+\exp\left(-\sum_{m=1}^{c^{\prime }}W_{6}\left[k_{2},m\right]F_{in}\left[b,m,i,j\right]+\sum_{m=1}^{c^{\prime }}W_{7}\left[k_{2},m\right]F_{in}^{\prime \prime }\left[b,m,i,j\right]\right)}$$

As defined in Equation (8), the spatial attention weight $W_{sp}\left[b,0,i,j\right]$ is then applied to $F_{ch}^{l}\left[b,k_{2},i,j\right]$ via element-wise multiplication to obtain the final output of the Bottleneck_DFF module, denoted as $F_{out}$. Here, ⊙ denotes element-wise multiplication:

(8) $$F_{out}=W_{sp}⊙F_{ch}^{l}=W_{sp}\left[b,0,i,j\right]F_{ch}^{l}\left[b,k_{2},i,j\right]\;$$

Outputs from multiple Bottleneck_DFF modules, together with the initial residual feature $F_{0}^{\prime }\left[b,k_{2},i,j\right]$, are concatenated using the Concat operation to form the aggregated feature map $F_{cat}\left[b,m,i,j\right]\in R^{B\times \left(n+1\right)C^{\prime }\times H\times W}$, where *n* is the number of Bottleneck_DFF modules, as formulated in Equation (9):

(9) $$F_{cat}\left[b,m,i,j\right]=\mathrm{Conact}\left(F_{0}^{\prime },F_{1},F_{2},\ldots \ldots ,F_{n}\right)$$

Finally, a convolution layer with a kernel $W_{8}\left[k_{3},m\right]$ is applied to produce the final output $Y_{cf}\in R^{B\times C_{out}\times H\times W}$, formulated in Equation (10). The kernel $W_{8}\left[k_{2},m\right]$, where $k_{3}\in \left[1,2,\ldots \ldots ,C_{out}\right]$, projects the concatenated features to the desired output channel dimension $C_{out}$:

(10) $$Y_{cf}\left[b,k_{3},i,j\right]=\overset{\left(n+1\right)c^{\prime }}{\underset{m=1}{\sum }}W_{8}\left[k_{3},m\right]F_{cat}\left[b,m,i,j\right]$$

#### 3.2.2. Multi-Scale Semantic Segmentation Branch

Object detection localises lesions at the object level but does not describe lesion shape or boundaries in detail. To complement detection, we add a semantic segmentation branch that provides pixel-level segmentation of lesion extent. The branch builds on YOLOv8 core modules and is inspired by multi-scale perceptual parsing in Unified Perceptual Parsing Network (UPerNet) [29], with additional design cues from the segmentation branch of YOLO-FD.

**Figure 4:**
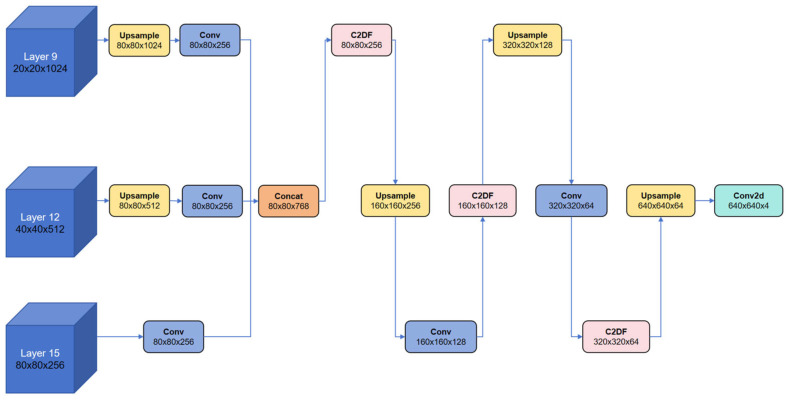
Seghead processing flowchart. Tensor dimensions are annotated beneath modules as height (H) × width (W) × channels (C).

We extract multi-layer features with varying semantic depths from the Backbone and Neck to serve as input for the segmentation branch. After spatial alignment and channel compression, we fuse the features to balance global semantic understanding and local edge information recovery. This fusion process enhances the model’s ability to model complex lesion regions. As shown in Figure 4, the segmentation branch selects feature maps from layers 9, 12, and 15 as input, representing high semantic, balanced, and high spatial resolution features, respectively. Specifically, the ninth layer provides the strongest semantic representations, making it ideal for modelling the global semantic structure of lesion regions. The 12th layer captures a balanced combination of semantic and spatial information, bridging global understanding and fine-grained detail. In contrast, the 15th layer retains the richest edge and texture details, crucial for accurately extracting lesion contours.

To ensure effective alignment and fusion of multi-scale feature maps, as shown in Figure 4, the 20 × 20 × 1024 feature map from the ninth layer is first upsampled by a factor of four and passed through a convolutional layer to reduce the channel dimension to 256, resulting in an 80 × 80 × 256 feature map. The 40 × 40 × 512 feature map from the 12th layer is upsampled by a factor of two and compressed to 256 channels, which results in a second feature map of the exact resolution. The feature map from the 15th layer is inherently 80 × 80 × 256. As a result, the three feature maps are spatially and dimensionally aligned, enabling efficient and seamless fusion via the Concat operation.

The decoder adopts a hierarchical upsampling strategy. Each stage comprises an upsampling layer, a Conv for refinement, and a C2DF block to enhance feature interactions. This progression restores resolution from 80 × 80 to 160 × 160, 320 × 320, and 640 × 640. A final Conv produces a four-channel output corresponding to three disease classes and background, yielding masks. On our dataset, the segmentation branch contributes to clearer boundary depiction and stable multi-scale responses, and it works jointly with the detection head within the multi-task framework.

### 3.3. Multi-Task Optimisation Strategy

In various fish disease detection and segmentation tasks, detection tasks focus on target localization and classification, while segmentation tasks focus on pixel-level region division. These two types of tasks differ regarding task objectives, feature focus, and gradient propagation direction. These differences can lead to loss imbalance, gradient conflicts, and task competition during multi-task joint training, weakening the overall model performance. This paper introduces weight uncertainty and PCGrad into FDMNet to mitigate these issues. Weight uncertainty can balance the convergence speed of multi-task learning in the loss function, while PCGrad can reduce optimisation interference between tasks in the gradient direction. The synergistic effect of the two improves the stability and performance of FDMNet.

#### 3.3.1. Weight Uncertainty

Following Kendall et al. [30], we use uncertainty-based multi-task weighting to balance detection and segmentation losses. The method maximises the joint likelihood across tasks, yielding an adaptive loss with one learnable uncertainty parameter per task. During training, these scalars are learned jointly with the network parameters, so the effective loss weights adapt to task difficulty rather than being fixed a priori.

Let the input image be x and the model parameters be w. The detection head prediction is denoted by $f^{w}\left(x\right)$. The detection target is $y_{1}$. The scalar $\sigma_{1}$ denotes the task uncertainty for detection. As shown in Equation (11), we model detection regression with a Gaussian whose mean is the model prediction and whose variance is ${\sigma_{1}}^{2}$:

(11) $$p\left(y_{1}|x,w,\sigma_{1}\right)=N\left(y_{1};f^{w}\left(x\right),{\sigma_{1}}^{2}\right).$$

For semantic segmentation, the target is $y_{2}$. The segmentation score map is denoted by $g^{w}\left(x\right)$. The scalar $\sigma_{2}$ denotes the task uncertainty for segmentation. In the uncertainty model, we scale the input to the Softmax by $\frac{1}{\sigma_{2}^{2}}$. The per-class likelihood is written as in Equation (12). In practice, this is applied per pixel:

(12) $$p\left(y_{2}|x,w,\sigma_{2}\right)=\mathrm{Softmax}\left(\frac{1}{\sigma_{2}^{2}}g^{w}\left(x\right)\right).$$

Assuming conditional independence across tasks given $\left(x,w\right)$, the joint likelihood factorises as in Equation (13):

(13) $$p\left(y_{1},\ldots ,y_{k}|x,w\right)=p\left(y_{1}|x,w\right)\ldots p\left(y_{k}|x,w\right).$$

Combining the Gaussian likelihood in Equation (11) with the Softmax likelihood in Equation (12) for the two-task case, Equation (14) gives:

$$p\left(y_{1},y_{2}|x,w,\sigma_{1},\sigma_{2}\right)=p\left(y_{1}|x,w,\sigma_{1}\right)\cdot p\left(y_{2}|x,w,\sigma_{2}\right)\;$$

(14) $$=\;N\left(y_{1};f^{w}\left(x\right),{\sigma_{1}}^{2}\right)\cdot \;\mathrm{Softmax}\left(\frac{1}{\sigma_{2}^{2}}g^{w}\left(x\right)\right)$$

By maximum likelihood, we minimise the negative log-likelihood in Equation (15):

(15) $$L\left(W,\sigma_{1},\sigma_{2}\right)=-\log p\left(y_{1},y_{2}|x,w,\sigma_{1}.\sigma_{2}\right)=-\log p\left(y_{1}|x,w,\sigma_{1}\right)-\log p\left(y_{2}|x,w,\sigma_{2}\right)$$

Expand the joint loss function $L\left(W,\sigma_{1},\sigma_{2}\right)$ to obtain the final multi-task total loss function. As shown in Equation (16), $L_{1}\left(W\right)$ is the detection task loss, and $L_{2}\left(W\right)$ is the segmentation task loss:

$$\left(W,\sigma_{1},\sigma_{2}\right)=\frac{1}{2\sigma_{1}^{2}}{\left\|y_{1}-f^{w}\left(x\right)\right\|}^{2}+\log\sigma_{1}-\frac{1}{\sigma_{2}^{2}}\log\mathrm{Softmax}\left(y_{2},f^{w}\left(x\right)\right)+\log\sigma_{2}\;\;$$

(16) $$\approx \frac{1}{2\sigma_{1}^{2}}L_{1}\left(W\right)+\frac{1}{\sigma^{2}}L_{2}\left(W\right)+\log\sigma_{1}+\log\sigma_{2}\;$$

The total loss function consists of the detection task loss $L_{det}$ and the segmentation task loss $L_{seg}$. The detection loss is a weighted combination of $L_{cls}$ (classification loss), $L_{iou}$ (bounding box regression loss), and $L_{dfl}$ (distributed regression loss). Equation (17) shows the detection loss, where *α*_1_ = 0.5, *α*_2_ = 7.5, and *α*_3_ = 1.5, all of which are conventional default values.

(17) $$L_{det}=\alpha_{1}L_{cls}+\alpha_{1}L_{iou}+\alpha_{3}L_{dfl}$$

The multi-task total loss function is defined in Equation (18):

(18) $$L_{all}=\frac{1}{2\sigma_{1}^{2}}L_{det}+\frac{1}{\sigma_{2}^{2}}L_{seg}+\log\sigma_{1}+\log\sigma_{2}$$

#### 3.3.2. PCGrad

In multi-task learning, detection and segmentation tasks are combined with different objectives. It is common for gradients generated during backpropagation across different tasks to cancel each other out, leading to issues such as hindered model optimisation and slow task convergence. To address this issue, this paper introduces the PCGrad algorithm proposed by Yu et al. [31]. At each backpropagation step, the algorithm computes per-task gradients and checks for conflicts. When a task’s gradient is misaligned with others, PCGrad adjusts that gradient via orthogonal projection to avoid interfering with the optimisation direction of the other tasks. As shown in Equation (19), let $g_{i}$ denote the gradient for the object-detection loss and $g_{j}$ the gradient for the semantic-segmentation loss. Their cosine similarity is:

(19) $$\cos\theta_{ij}=\frac{g_{i}\cdot g_{j}}{|\left|g_{i}\right|{|}^{2}}$$

When $\cos\theta_{ij}<0$, the two task gradients are in conflict. PCGrad projects $g_{i}$ to remove its component along $g_{j}$:

(20) gi′=gi−gi·gj|gi|2 gj 

If $\cos\theta_{ij}\geq 0$ indicates no conflict between gradients, and PCGrad will keep the original gradient unchanged. Unlike the uncertainty weighting mechanism, which adjusts the task contributions from the perspective of task loss scale, PCGrad focuses on gradient direction optimisation.

## 4. Results

### 4.1. Experimental Details

All models were trained for 200 epochs on 2148 images, with 640 × 640 inputs and batch size 8, using a single RTX 4070 GPU (Intel, Santa Clara, CA, USA). Training used automatic mixed precision (AMP) and a fixed random seed (11) with deterministic flags for reproducibility. The initial uncertainty parameters in the loss, $\sigma_{1}$ and $\sigma_{2}$, were set to 10.689 and 0.17. The system configuration is summarised in Table 2.

Optimisation used AdamW with an initial learning rate of 4.7 × 10^−4^, cosine decay to 1% of the initial value over 200 epochs, with a 3-epoch warm-up. The exponential decay rates were β_1_ = 0.937 and β_2_ = 0.999, and the weight decay was 5 × 10^−4^. This configuration yields stable convergence and good performance on a held-out in-distribution test set.

To mitigate overfitting, we applied data augmentation to reduce the model’s memorization of sample-specific details. The data augmentation comprised HSV jitter (h = 0.015, s = 0.7, v = 0.4), random translation (±0.1 of the image size), random scaling (±0.5), and horizontal flip (*p* = 0.5). Mosaic augmentation was enabled for most training and disabled during the final 12 epochs to stabilise optimisation. Additionally, we used a MixUp ratio of 0.1. We applied weight decay (5 × 10^−4^) to penalise large weights and constrain effective model capacity. Finally, we used a held-out validation set with early stopping (patience = 30) based on validation mAP. We maintained an exponential moving average (EMA) of the parameters (decay = 0.9999) to smooth updates and improve generalisation.

### 4.2. Experimental Results

#### 4.2.1. Evaluation Metrics

We evaluate experimental results using established metrics: Precision, Recall, mAP50, and mIoU. This choice follows common practice and enables direct comparison with widely used models. Precision measures the proportion of predicted positives that are correct. It reflects sensitivity to false positives. Recall measures the proportion of ground-truth objects that are detected. It reflects the risk of missed detections. Using Precision and Recall together makes the trade-off between false positives and false negatives explicit. The metric mAP50 is the mean Average Precision computed at an IoU threshold of 0.5. It averages this value across classes, summarising detection accuracy and localization at a fixed overlap threshold.

As shown in Equations (21), the calculation formulas for Precision and Recall are as follows. A prediction is a true positive (TP) if its intersection over union with the best matching ground truth is at least 0.5. Unmatched predictions are false positives (FP), and unmatched ground truths are false negatives (FN):

$$\;\mathrm{Precision}=\;\frac{\mathrm{TP}}{\mathrm{TP}+\mathrm{FP}}\;\;\;\;\;$$

(21) $$\;\mathrm{Recall}=\frac{\mathrm{TP}}{\mathrm{TP}+\mathrm{FN}}$$

For each class k, Average Precision ${\mathrm{AP}}_{k}$ is the area under the precision–recall curve at IoU = 0.5. With K classes,

(22) $$\;\mathrm{mAP}50=\frac{1}{K}\overset{K}{\underset{k=1}{\sum }}{\mathrm{AP}}_{k}$$

For segmentation, we report mean Intersection over Union (mIoU). For class k,

(23) $${\;\mathrm{IoU}}_{k}=\frac{{\mathrm{TP}}_{k}}{{\mathrm{TP}}_{k}+{\mathrm{FP}}_{k}+{\mathrm{FN}}_{k}}\;$$

where $TP_{k}$, $FP_{k}$ and $FN_{k}$ are pixel counts for class k. The mean IoU is

(24) $$\;\;\mathrm{mIoU}=\frac{1}{K}\overset{K}{\underset{k=1}{\sum }}{\mathrm{IoU}}_{k}\;$$

In this study, K=3 disease classes (excluding background). All metrics were computed on the held-out test set.

#### 4.2.2. Object Detection Results

To assess FDMNet’s effectiveness in object detection, we compared it with representative detectors: Faster R-CNN, YOLOv8n and YOLOv11n, Transformer-based RT-DETR, and the multi-task models YOLO-FD and Mask R-CNN. Evaluation metrics include precision, recall, mAP50, parameter count, and per-image speed. The results are summarised in Table 3.

On our dataset, FDMNet achieves the highest overall detection performance among the compared methods, with a precision of 95.3%, a recall of 92.1%, and an mAP50 of 97.0%. Relative to the YOLOv8n baseline, FDMNet improves precision by 3.9%, recall by 1.4%, and mAP50 by 3.4%. FDMNet adds just 0.1 M parameters over YOLOv8n and supports semantic segmentation. RT-DETR attains the highest precision (96.1%) but exhibits substantially lower recall, with a larger parameter budget and longer latency, which limits its suitability for real-time and resource-constrained deployments. Compared with Mask R-CNN and YOLO-FD, FDMNet achieves higher precision, recall, and mAP50. While its parameter count is slightly larger than YOLO-FD, improving key accuracy metrics may justify this trade-off for accuracy-sensitive scenarios. Overall, FDMNet provides a favourable accuracy–efficiency balance for fish disease detection and indicates potential for practical deployment within the evaluated conditions.

**Figure 5:**
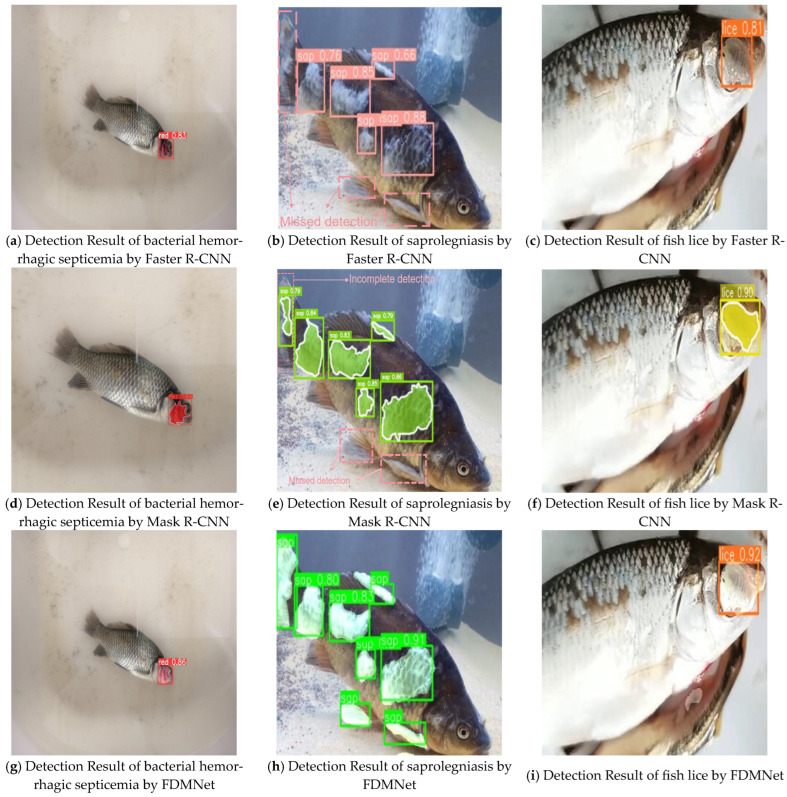
Qualitative comparison of detection results on the held-out test set for three fish diseases—bacterial hemorrhagic septicemia, saprolegniasis, and fish lice—using Faster R-CNN, Mask R-CNN, and FDMNet. Panels map as follows: (**a**,**d**,**g**) bacterial hemorrhagic septicemia, (**b**,**e**,**h**) saprolegniasis, (**c**,**f**,**i**) fish lice, where (**a**–**c**) are Faster R-CNN, (**d**–**f**) are Mask R-CNN, and (**g**–**i**) are FDMNet. All models were evaluated under the same protocol on the same test split at an input resolution of 640 × 640. Visualisations use a confidence threshold of 0.5. Bounding boxes and mask overlays are shown as applicable. Colour legend: red mask for bacterial hemorrhagic septicemia, green mask for saprolegniasis, and yellow mask for fish lice.

All models were trained under the same input resolution and evaluated on our dataset. The best checkpoint for each model was selected on the validation performance and then evaluated on the test set. Figure 5 presents qualitative comparisons for bacterial hemorrhagic septicemia, saprolegniasis, and fish lice using Faster R-CNN, Mask R-CNN, and FDMNet. For saprolegniasis, the single-task detector Faster R-CNN often yields incomplete detections, particularly on the tail and fins. The multi-task Mask R-CNN improves coverage and detects tail lesions, yet misses portions of the affected regions. In these examples, FDMNet provides more complete detections across the tail and fin areas.

#### 4.2.3. Semantic Segmentation Results

Classic semantic segmentation networks, including U-Net, DeepLabv3-ResNet50, and DeepLabv3+-ResNet50, were chosen to compare with the proposed model. We also evaluated lightweight DeepLabv3 and DeepLabv3+ variants with MobileNet backbones to keep parameter counts comparable to FDMNet. In addition, we included YOLO-FD and Mask R-CNN as multi-task comparison models. We report mIoU, parameter count, FLOPs, and speed in Table 4. On our dataset, FDMNet attains the highest mIoU. Relative to U-Net, DeepLabv3-ResNet50, DeepLabv3+-ResNet50, Mask R-CNN, and YOLO-FD, the mIoU gains are 20.9%, 12.3%, 9.0%, 16.2%, and 5.4%. While FDMNet’s parameter count and latency are slightly higher than YOLO-FD, the overall accuracy–efficiency trade-off remains favourable for practical deployment. These quantitative results support the effectiveness of joint detection–segmentation training for this application while maintaining a compact model design.

**Figure 6:**
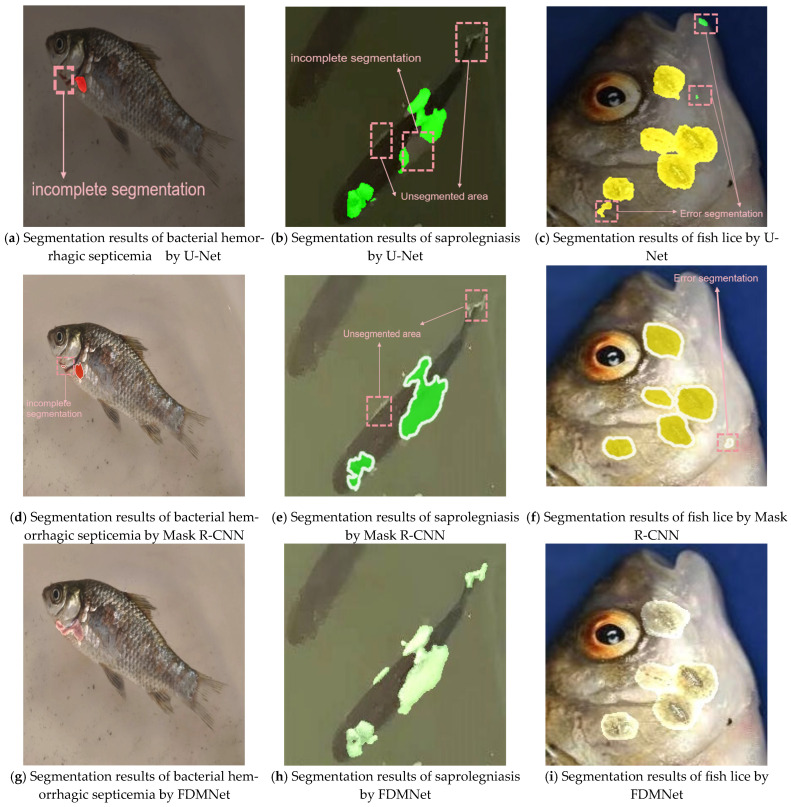
Qualitative comparison of semantic segmentation on the same test images for three fish diseases—bacterial hemorrhagic septicemia, saprolegniasis, and fish lice—using U-Net, Mask R-CNN, and FDMNet. Panels map as follows: (**a**,**d**,**g**) bacterial hemorrhagic septicemia, (**b**,**e**,**h**) saprolegniasis, (**c**,**f**,**i**) fish lice, where (**a**–**c**) are U-Net, (**d**–**f**) are Mask R-CNN, and (**g**–**i**) are FDMNet. All models were evaluated under the same protocol on the held-out test split at an input resolution of 640 × 640. red mask = bacterial hemorrhagic septicemia, green = saprolegniasis, yellow = fish lice.

Figure 6 provides qualitative comparisons among U-Net, Mask R-CNN, and FDMNet. Across the three diseases—bacterial hemorrhagic septicemia, saprolegniasis, and fish lice—FDMNet produces more complete masks and more precise lesion segmentation in these examples. U-Net and Mask R-CNN show task-specific limitations. For bacterial hemorrhagic septicemia, both tend to under-segment small lesion regions. For saprolegniasis, they frequently miss portions of tail- and fin-area lesions. For fish lice, they exhibit noticeable boundary errors and fragmented masks. By contrast, FDMNet more consistently localises lesions and yields accurate, contiguous segmentations across the three conditions on the same images.

### 4.3. Ablation Experiments

To investigate the coupling effects between detection and segmentation tasks in multi-task learning, we compare single-task training (detection-only or segmentation-only) with joint multi-task training. As shown in Table 5, the results demonstrate consistent performance improvements across all metrics when using multi-task learning, regardless of whether the C2f or C2DF module was employed. Specifically, multi-task training yielded higher detection precision, mAP50, and mIoU values than single-task approaches. These findings suggest a synergistic relationship between object detection and semantic segmentation, where each task enhances the other’s performance. The segmentation task contributes by refining the model’s understanding of spatial structures and semantic features through pixel-level boundary analysis. In contrast, the detection task provides precise object localization, improving segmentation boundary accuracy.

Replacing C2f with C2DF during multi-task training yields additional gains. Relative to the C2f variant, precision increases from 0.933 to 0.953, recall from 0.898 to 0.921, mAP50 from 0.945 to 0.970, and mIoU from 0.823 to 0.857. These increases suggest that C2DF provides improved representational capacity for joint detection–segmentation under our settings. In our setup, Detection-only models train only the detection head and do not report mIoU. Segmentation-only models train only the segmentation branch and do not report detection metrics. Non-applicable metrics are marked with “—”.

To compare the attention patterns of C2DF with those of the original C2f for lesion-relevant features, we select layer 15 in the detection head as the site for heat map analysis. This layer aggregates fine-grained information from lower levels. It is informative for small targets and edge-sensitive cues, common in fish disease lesions characterised by small size, complex morphology, and blurred boundaries.

**Figure 7:**
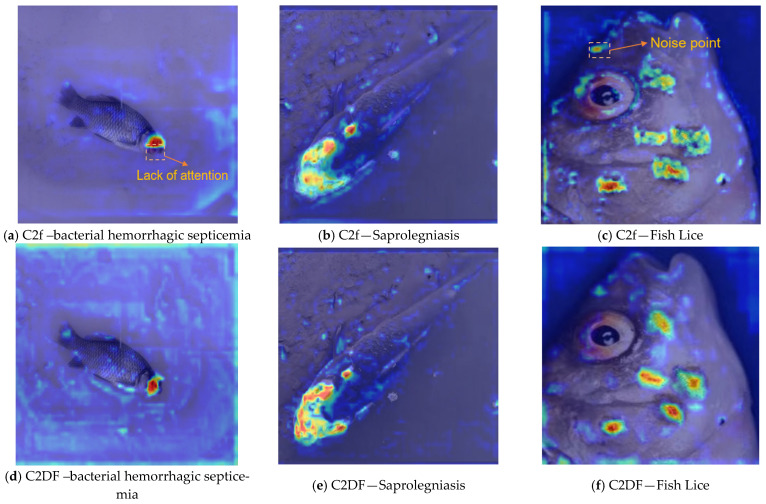
Attention heatmaps comparing the C2f and C2DF variants on the same test images for three fish diseases: bacterial hemorrhagic septicemia, saprolegniasis, and fish lice. Panels map as follows: (**a**–**c**) C2f variant and (**d**–**f**) C2DF variant; columns correspond to (**a**,**d**) bacterial hemorrhagic septicemia, (**b**,**e**) saprolegniasis, and (**c**,**f**) fish lice. Heatmaps are computed with Grad-CAM on the P3 detection-head feature (Figure 2, layer 15; 80 × 80, stride 8). All visualisations use the same test split, input resolution 640 × 640.

We apply Grad-CAM to the output of Layer 15 to display how C2DF and C2f attend to lesion regions. As shown in Figure 7, the C2f-based maps sometimes provide insufficient coverage of diseased areas for bacterial hemorrhagic septicemia and exhibit spurious activations for fish lice. In these examples, FDMNet with C2DF produces more contiguous activations that align more closely with the annotated lesions across all three diseases. These observations are consistent with the improved feature separation and fine-grained sensitivity afforded by C2DF and complement the quantitative gains reported in Table 5.

## 5. Discussion

### 5.1. Model Evaluation

FDMNet achieves consistent detection and segmentation performance, providing an integrated solution for multi-task fish disease analysis. On our dataset, FDMNet achieved 97.0% mAP50 for detection and 85.7% mIoU for segmentation. Relative to the multi-task baseline YOLO-FD, FDMNet improved mAP50 by 2.5% and mIoU by 5.4% under the same evaluation protocol, while maintaining a compact parameter count and low single-image latency on our setup. These gains are attributable to two design choices: a multi-scale segmentation branch that aggregates features across semantic levels for small, diffuse, or boundary-sensitive lesions; and the C2DF module, which combines channel and spatial attention to enhance feature separation and fine-grained perception. In addition, coupling uncertainty-based loss weighting with PCGrad mitigates inter-task gradient conflicts and stabilises joint training. Although FDMNet incurs a modest increase in latency and parameters compared with the lightest baselines, the resulting accuracy–efficiency trade-off aligns with practical deployment constraints.

### 5.2. Model Limitations and Future Work

Although FDMNet achieves favourable results on our dataset, it has more parameters and higher inference latency than the lightest baselines. This overhead is mainly due to the added segmentation branch. It may make deployment more difficult on edge devices with limited resources. Early in training, fusing low-level features can misalign multi-scale representations. This misalignment produces noisy or inconsistent gradients and destabilises training, especially near ambiguous lesion boundaries. The evaluation scope is limited to a random split of a single dataset, so the reported results reflect within-distribution performance only. Robustness under distribution shift has not yet been assessed. We did not conduct out-of-distribution validation across different farms, imaging modalities, or acquisition settings. In addition, the current dataset does not include examples of fish with more than one disease. This absence may restrict performance in real-world scenarios where multi-disease cases occur.

Future work will expand disease coverage beyond the three categories to include additional common fish diseases. Data collection will also be broadened across species, farms, devices, and acquisition conditions. We will systematically collect cases where a single fish has multiple diseases, with multi-label annotations at the image, object, and pixel levels. We will evaluate cross-domain generalisation and report both in-distribution and out-of-distribution performance. On the architectural side, we will explore more efficient cross-scale alignment and fusion, employ lightweight attention, pruning, quantisation, and knowledge distillation, and assess on-device latency and throughput to target a favourable accuracy–efficiency trade-off. For learning stability, we will investigate curriculum or warm-up fusion, uncertainty-aware loss reweighting, and strategies to mitigate gradient conflicts. Beyond vision, we aim to integrate sonar, ultrasound, infrared thermography, and environmental pressure sensing, examining early and late fusion, robustness to missing modalities, and sensor synchronisation. We will also pursue model calibration and uncertainty estimation, human-in-the-loop triage for low-confidence cases, periodic retraining, and on-site validation to improve reliability and facilitate deployment in resource-constrained aquaculture systems.

## 6. Conclusions

This study presents FDMNet, a lightweight multi-task framework built on YOLOv8n. The model adds a semantic segmentation branch and the C2DF dynamic feature-fusion modules to jointly perform detection and lesion segmentation for three common fish diseases. The training strategy combines uncertainty-based loss weighting with PCGrad to reduce conflicts between tasks and improve training stability. Together, these components provide an integrated pipeline for image-level detection and pixel-level segmentation while retaining a compact parameter budget.

On the held-out test split of our in-house dataset, FDMNet achieved 97.0% mAP50 for detection and 85.7% mIoU for segmentation, with improvements over representative single-task and multi-task baselines. Compared with YOLO-FD, improvements were 2.5% in mAP50 and 5.4% in mIoU. Ablation analyses indicate synergistic benefits of multi-task training and additional improvements from C2DF. Overall, the proposed architecture offers a favourable accuracy–efficiency trade-off under the evaluated conditions.

## Figures and Tables

**Table 1:** Dataset characteristics and annotation statistics.

Disease Type	Images	Train	Validation	Test	Objects	Masks
bacterial hemorrhagic septicemia	528	370	106	52	989	1002
saprolegniasis	512	358	102	52	913	925
fish lice	508	355	102	51	907	919
Healthy	600	420	120	60	0	0
Total	2148	1503	430	215	2809	2846

Notes. “Disease type” lists the three disease categories considered. “Healthy” is included only as a negative class to model background and reduce false positives and is not treated as a fourth disease. Images: number of labelled images per class (classes are mutually exclusive and sum to the total). Train/Validation/Test: image counts per split using an approximately 7:2:1 ratio (rounded to whole images). Objects: total number of annotated detection boxes in YOLO-format txt files across all splits. Masks: total number of polygon instances in LabelMe annotations across all splits (a single image can contribute multiple masks).

**Table 2:** Experimental configuration.

Configuration	Parameter
CPU	12th Gen Intel Core i7-12800HX (Intel, Santa Clara, CA, USA)
GPU	NVIDIA GeForce RTX 4070
Operating system	Windows 11
Accelerated environment	CUDA Toolkit: 12.4
Development environment	Visual Studio Code
Deep learning framework	PyTorch 2.5.0

**Table 3:** Comparison of FDMNet with representative object detection models.

Algorithms	Precision	Recall	mAP50	Parameters (M)	Speed
Faster R-CNN	0.742	0.694	0.834	41.3 M	9.6 ms
YOLOv8n	0.914	0.907	0.936	3.2 M	3.8 ms
YOLOv11n	0.892	0.855	0.928	2.59 M	4.3 ms
RT-DETR	0.961	0.770	0.961	43.7 M	14.5 ms
Mask R-CNN	0.762	0.709	0.831	37.7 M	65.9 ms
YOLO-FD	0.933	0.898	0.945	3.23 M	5.9 ms
FDMNet	0.953	0.921	0.970	3.33 M	6.8 ms

**Table 4:** Segmentation performance comparison of FDMNet with baseline models.

Algorithms	mIoU	Params	FLOPs	Speed
U-Net	0.648	4.3 M	40.1 G	18.6 ms
Deeplabv3-MobileNet	0.719	5.1 M	5.8 G	7.4 ms
Deeplabv3-ResNet50	0.734	39.6 M	51.1 G	23.5 ms
Deeplabv3+-MobileNet	0.751	5.2 M	16.8 G	8.0 ms
Deep-labv3+-ResNet50	0.767	39.9 M	62.4 G	28.1 ms
Mask R-CNN	0.695	37.7 M	95.7 G	65.9 ms
YOLO-FD	0.803	3.23 M	14.7 G	5.9 ms
FDMNet	0.857	3.33 M	15.1 G	6.8 ms

**Table 5:** Results of the ablation experiment.

Algorithms	Precision	Recall	mAP50	mIoU
Det only	0.914	0.907	0.936	—
Seg only	—	—	—	0.808
Multi-task (C2f)	0.933	0.898	0.945	0.823
Multi-task (C2DF)	0.953	0.921	0.970	0.857

## Data Availability

The data presented in this study are available on request from the corresponding author.
